# Improving the Reliability of Literature Reviews: Detection of Retracted Articles through Academic Search Engines

**DOI:** 10.3390/ejihpe12050034

**Published:** 2022-05-04

**Authors:** Elena Pastor-Ramón, Ivan Herrera-Peco, Oskia Agirre, María García-Puente, José María Morán

**Affiliations:** 1 Virtual Health Sciences Library of the Balearic Islands (Bibliosalut), 07120 Palma, Spain; epastor@bibliosalut.com; 2 Faculty of Communication, Pompeu Fabra University, 08018 Barcelona, Spain; iherrpec@uax.es; 3 Nursing Department, Faculty of Medicine, Universidad Alfonso X el Sabio, 28621 Madrid, Spain; 4 Public Health and Paharmacy Department, Faculty of Health Sciences, Universidad Alfonso X el Sabio, 28621 Madrid, Spain; oskiamiren.aguirreorbegozo@osakidetza.eus; 5 Osakidetza, Basque Health Service, Araba Integrated Health Organisation, Araba University Hospital, Jose Atxotegi Kalea, s/n, 01900 Vitoria-Gasteiz, Spain; 6 Faculty of Social and Communication Sciences, Doctoral Programme in Social Communication, University of the Basque Country UPV/EHU, 48940 Leioa, Spain; 7 Fundación Jiménez Díaz, 28040 Madrid, Spain; maria@alterbiblio.com; 8 Metabolic Bone Diseases Research Group, Nursing Department, Nursing and Occupational Therapy College, University of Extremadura, 10003 Caceres, Spain

**Keywords:** research methodology, biomedical publishing, publication ethics, scientific misconduct, retraction of publication

## Abstract

Nowadays, a multitude of scientific publications on health science are being developed that require correct bibliographic search in order to avoid the use and inclusion of retracted literature in them. The use of these articles could directly affect the consistency of the scientific studies and could affect clinical practice. The aim of the present study was to evaluate the capacity of the main scientific literature search engines, both general (Gooogle Scholar) and scientific (PubMed, EMBASE, SCOPUS, and Web of Science), used in health sciences in order to check their ability to detect and warn users of retracted articles in the searches carried out. The sample of retracted articles was obtained from RetractionWatch. The results showed that although Google Scholar was the search engine with the highest capacity to retrieve selected articles, it was the least effective, compared with scientific search engines, at providing information on the retraction of articles. The use of different scientific search engines to retrieve as many scientific articles as possible, as well as never using only a generic search engine, is highly recommended. This will reduce the possibility of including retracted articles and will avoid affecting the reliability of the scientific studies carried out.

## 1. Introduction

Obtaining scientific knowledge is based on scientific methods, which can be defined as the specific protocols that serve as a guide in the process of observing reality, an observation that in turn will allow for knowledge to be acquired. However, it is important to point out that this process does not end with the dissemination of the findings, although it may seem so, as the publication has already been reviewed by scientific peers, which ensures, a priori, its scientific quality [1,2]. Rather, it is the task of the scientific community to review knowledge that has already been published with a critical eye in order to identify possible situations that could lead to situations wherein the knowledge produced has not been obtained in a methodologically appropriate manner [3].

The outcome of these post-review processes, essential in the self-correcting approach in science [4], may result in finding some situations that may be associated with scientific misconduct, such as (i) a compromised peer review process, (ii) duplication of publication, (iii) duplication of images, (iv) lack of ethical approval, (v) plagiarism, and (vi) undeclared conflicts of interest, among others [5,6]. 

However, at first glance, this situation may seem anecdotal; the number of retracted scientific publications has increased enormously, from less than 100 in 2000 to 1772 reported in 2019 [7]. Analyzing the RetractionWatch database as a reference where retracted articles are registered [8], it was found that in the period from 31 December 2019, to 25 December 2021, 2615 article retraction notes have been published [9]. The data show that the number of retracted articles has significantly increased in the last ten years [10].

Although this scientific literature is retracted and appears as such in numerous databases, it has been found that these articles continue to be cited [11]. In some instances, correct citations are given, indicating that the article has been retracted; however, in other instances, articles are usually cited as if they have not been retracted [10,12]. The continuous citation of retracted articles likely comes from an inadequate process when selecting references in studies. This situation may be due to (i) citing secondary sources and using the copy-and-paste method from one article to cite others [6] and (ii) not appropriately using scientific browsers and other tools to identify the retracted articles [3]. The first point relates to scientific praxis and the need to use primary sources. Regarding the second point, there are search engines that clearly show whether an article is retracted, such as Web of Science [12] or PubMed [3]. There are also other methods, known as automated citation checking services, such as scite.ai [13], Zotero [14], and RedacTek [14]; failing to use these tools is a disservice to readers and researchers. The problem raised was identified through the use of SCRUTATIOm [14], a rapid, reproducible, and systematic method for detecting retracted literature included in research studies, which allows for the possibility of communicating the possible presence of flaws to the scientific community through a post-publication or post-peer-review process. The procedure is based on the combined use of the Scopus database and the Zotero bibliographic reference manager through a five-step process.

The main problem with the continued use of retracted literature is that the incorporation of these invalid studies into literature reviews can potentially distract future research and clinical attention [15,16,17,18]. Harold Sox and Drummond Rennie [19] outlined the responsibilities of institutions, editors, and authors who cite literature to prevent the continued citation of fraudulent research. Authors submitting manuscripts for publication are responsible for checking the references cited in their bibliography to see if they have been retracted, and authors (or readers) who detect that a published paper refers to a retracted article are also responsible for submitting a correction to the journal [18,19].

Based on the above, the following study was designed to analyze the capacity of these search engines to provide results that allow for the identification of retracted literature. The aim of the present study is to compare some of the most widely used scientific search engines in the health sciences, and to compare these search engines to find out their success rate in the recognition of retracted articles and how they warn users of this situation. The main hypothesis is that general search engines will offer lower recognition rates than those considered to be scientific. Regarding this objective, the research hypothesis is that general search engines will offer lower recognition rates of retracted articles than scientific search engines.

## 2. Materials and Methods

### 2.1. Data Collection

This comparative study was based on a search of the RetractionWatch database conducted on 26 December 2021, for retracted articles, where the original manuscript was published between 1 January 2016, and 25 December 2021.

We selected (i) all types of articles and (ii) all reasons for retraction, and (iii) the selected subject being searched was “diabetes”. Finally, we obtained a total of 50 retracted articles, which can be found in the Supplementary Material.

In order to check the reliability of RetractionWatch, so as to ensure that the articles retrieved from RetractionWatch were really retracted, it was verified that these articles appeared as such on the web page corresponding to each of the journals in which they were published.

The search engines selected for this study were (i) PubMed, (ii) Scopus, (iii) Web of Science, (iv) EMBASE, and (v) Google Scholar, all of which are widely used in health sciences. Both PubMed and Google Scholar allow for a free and direct search of their content. PubMed offers access to articles about medicine and biomedical sciences, indexed in Medline and PubMed Central databases. Google Scholar covers most of the scientific fields through searching for articles indexed from publishers, libraries, repositories, or bibliographic databases [20]. Furthermore, Scopus, Web of Science, and EMBASE are resources widely used by researchers that cover most scientific fields [21,22], but access to their resources requires subscription by the researchers’ institutions.

In the process of reviewing the documents, two authors searched and reviewed the articles independently in each of the search engines selected. The results were analyzed according to the following criteria: (i) indexed or not indexed, and (ii) detected or not detected.

Here, indexed refers to articles found in journals indexed in databases accessed by the selected search engine. Furthermore, detected means that the search engine provided information on the retraction of the manuscript. In addition, not detected means that the search engine did not provide information on the retraction of the manuscript. In this study, we considered the information on the retraction when the search engines offered article information that indicated that it had been withdrawn or the original article was shown in conjunction with a retraction note as adequate. However, expression of concern was considered as not providing adequate notice to authors.

### 2.2. Data Analysis

The data analysis was performed using IBM-SPSS version 23.0 (IBM Corporation, New York, NY, USA). The descriptive statistics for categorical variables were presented as frequency (percentage); in order to identify the possible relation between variables, the Chi square test, a non-parametric analysis, was carried out. The statistical level of significance was set at *p* < 0.05.

## 3. Results

Firstly, the indexation capacity of the databases wherein the evaluated search engines found the articles was reviewed. In this case, it was found that the best search engine out of all of those used was Google Scholar, with search results offering access to 96% of the articles consulted, followed by PubMed (76%), EMBASE (72%), SCOPUS (68%), and WoS (56%) (Table 1).

The percentage was calculated in relation the total of indexed manuscripts for each search engine. 

Not indexed means that the databases wherein the search engine searches for the information did not include the journals where the manuscript was published.

Within the analysis associated with checking whether they offered information on whether or not an article had been retracted, it was found that PubMed showed 28 (73.68%) out of the total of 38 articles indexed in the databases searched by this search engine. The next search engine in terms of efficiency for showing retracted documents was Web of Science, with 18 out of a total of 28 indexed. The search engine that showed the worst results when it came to displaying information about retracted articles was Google Scholar, where only 15 out of 48 indexes showed information about the retraction of these articles.

In relation to the total number of documents analyzed, PubMed was the search engine with the best result, as 56% of the indexed documents showed information about their retraction, followed by EMBASE (42%), SCOPUS (36%), Web of Science (36%), and Google Scholar (30%).

The comparison between the different search engines showed that Google Scholar had a greater capacity to find articles than EMBASE (*p* < 0.0001), PubMed (*p* = 0.004), SCOPUS (*p* < 0.0000), and Web of Science (*p* < 0.0001). It was also observed that PubMed had a better ability to find and display selected articles than Web of Science (*p* = 0.035) (Table 2).

It was found that the ability to detect and warn researchers of retracted articles was significantly higher in all search engines compared with the ability shown by Google Scholar, in particular for PubMed (*p* = 0.0001). When comparing the rest of the search engines, no significant differences were observed in terms of their ability to detect and display information about retracted articles, within those that were indexed in the databases searched by these search engines (Table 3).

## 4. Discussion

While the process of retracting articles is a lengthy process, as it is complex to uncover possible problems of plagiarism or scientific misconduct [17], the main problem may come after the retraction has occurred, as the COPE guidelines suggest that it is only necessary that the retraction notice is open access, but does not indicate that access to the retracted article should be removed or made unavailable for consultation/access [17]. Because of the absence of a retraction notice removal of retracted articles, many journals still provide access, even in open access, which facilitates access by researchers who may not realize that this document has been retracted [11,12]. However, not only journals themselves but also databases and search engines continue to index them, so these articles, known as “zombie literature”, are still accessible [23,24,25], generating confusion and affecting clinical decision-making processes [3,26].

Furthermore, although it seems clear that any bibliographic search process for an academic study requires a meticulously structured and well-defined information retrieval process that requires several sources of information [26], it is also true that there is an increasing tendency to use a single scientific search engine to search for articles of interest [20]. In this sense, it is becoming increasingly common, especially with Google Scholar [20,23], that the use of a single search engine does not offer all of the coverage of articles that a proper bibliographic search requires [20]. However, it offers wide coverage in terms of the articles it can access, which is consistent with the findings in our study [20]. In addition, researchers are increasingly using pirate repositories, such as SciHub, as if they are databases that offer complete information on the articles included therein, producing a problem of reproducibility and reliability of their bibliographic searches, which affects the reliability of the review itself [27].

Our findings have shown that a single scientific search engine, whether open source or subscription-based, is not 100% effective at recognizing and providing notice to users that a document has been retracted, a situation that is consistent with the limitations that each search engine may have. For example, Google Scholar has been described as not allowing reproducible searches, as well as other problems derived from the automatic indexing process, such as (i) duplicity in records, (ii) ghost authors, and (iii) the inclusion of non-academic content, among others [28]. However, other search engines, free or not, have a common limitation, which is the coverage of the titles included in the databases in which the searches are carried out. For example, PubMed handles more than 30 million records [29], but it does not have all of the articles associated with the health sciences. SCOPUS searches journals (more than 23,000), conferences (120,000) and books (206,000), containing a total of about 77.8 million records [30], but only those indexed by Elsevier [30]. Web of Science offers only, in the Science Citation Index Expanded (SCIE), more than 9200 journals indexed by Thomson Reuters with 53 million records [30,31].

In summary, no database provides 100% coverage of the scientific production in a specific area, which makes it necessary to use several of them in order to obtain greater coverage of the information retrieved.

However, there are other tools that allow users to identify retracted articles, like databases that collect retracted articles and provide reasons for being retracted, with great reliability and that allow open access to this information, as is the case of RetractionWatch [8,14]. These tools, which are alternatives to those used in bibliographic searches, are not usually consulted by researchers.

In view of the above and given the real situation regarding the bibliographic search for scientific information, it is also necessary to use search engines that have notice-to-users’ tools focused on providing information about the reliability of the scientific articles they are consulting [14]. This situation is critical, as there may be situations in which authors have included retracted literature in systematic reviews or meta-analyses, which would affect the validity of their findings and conclusions [3]. Given that literature searches in the health sciences are often performed in certain search engines [21], it is important that they include warning systems for retracted literature. Presently, only PubMed and Google Scholar, through the Chrome browser application RetractOmatic, offer a visual warning that is easy to recognize for users.

This study has some limitations; first of all, a small number of articles derived from a specific topic were used. Secondly, it should also be noted that the database from RetractionWatch was used to collect retracted articles. This database, although it may not be considered exhaustive, is considered to be reliable because the documents indexed there are retracted.

## 5. Conclusions

We consider it essential to enhance training about the correct use of the location and selection of the most relevant scientific manuscripts, avoiding the inclusion of retracted articles that could compromise the scientific body of knowledge in health sciences.

Our intention is only to emphasize the importance of an accurate review of the literature and, especially, to bring to the attention of researchers the harm that is done to the body of knowledge when we include retracted literature in our studies, particularly when the reason is related to the integrity of data and results and scientific misconduct.

It is a recommended practice to use several scientific search engines that both allow access to many scientific articles and provide information on whether these can be considered as solid scientific evidence or, on the contrary, whether they have been retracted and should not be used in scientific studies.

Based on these findings, a study covering a longer period of time and a specific field of health sciences should be carried out in the future to compare in detail the efficiency and detection capacity of retracted documents of the main search engines used in the health sciences, as well as the analysis of reason for retraction.

## Figures and Tables

**Table 1:** Search engine capabilities for detected manuscripts, retracted selected.

Search Engine	Indexed			Not Indexed (*n*; %)
	Total (*n*; %)	Detected (*n*; %)	Not Detected (*n*;%)	
EMBASE	36; 72%	21; 58.33%	15; 41.67%	14; 28%
Google Scholar	48; 96%	15; 31.25%	33; 68.75%	2; 4%
PubMed	38; 76%	28; 73.68%	10; 26.32%	12; 24%
SCOPUS	34; 68%	18; 52.94%	16; 47.06%	16; 32%
Web of Science (WoS)	28; 56%	18; 64.29%	10; 35.71%	22; 44%

**Table 2:** Comparison of search engine capability to retrieve requested items.

	Embase	Google Sholar	PubMed	SCOPUS	Web of Science
Embase					
Google Scholar	21.93; <0.0001				
PubMed	0.208; 0.648	8.306; 0.004			
SCOPUS	3.894; 0.058	13.279; <0.0000	0.794; 0.373		
Web of Science	2.778; 0.096	21.39; <0.0001	4.456; 0.035	1.528; 0.216	

Date shown as $\left(X^{2}\;;p\right)$.

**Table 3:** Comparison between search engine capability to detect and show the retraction notice from the articles selected.

	Embase	Google Sholar	PubMed	SCOPUS	Web of Science
Embase					
Google Scholar	0.812; 0.368				
PubMed	3.347; 0.067	0.674; 0.412			
SCOPUS	3.894; 0.048	7.856; 0.005	15.216; 0.0001		
Web of Science	0.206; 0.65	0.234; 0.628	1.947; 0.163	6.161; 0.013	

Date shown as $\left(X^{2}\;;p\right)$.

## Data Availability

The data that support the findings of this study are available from the corresponding author upon reasonable request.

## Supplementary Materials

The following supporting information can be downloaded at: https://www.mdpi.com/article/10.3390/ejihpe12050034/s1.
